# Early Experience with Prone Lateral Interbody Fusion in Deformity Correction: A Single-Institution Experience

**DOI:** 10.3390/jcm13082279

**Published:** 2024-04-15

**Authors:** Alyssa M. Bartlett, Christopher F. Dibble, David A. W. Sykes, Peter N. Drossopoulos, Timothy Y. Wang, Clifford L. Crutcher, Khoi D. Than, Deb A. Bhomwick, Christopher I. Shaffrey, Muhammad M. Abd-El-Barr

**Affiliations:** 1 Department of Neurosurgery, Duke University, Durham, NC 27710, USA; alyssa.bartlett@duke.edu (A.M.B.);; 2 Department of Neurosurgery, Wake Forest University School of Medicine, Winston-Salem, NC 27157, USA

**Keywords:** minimally invasive spine surgery, interbody fusion, spinal deformity, prone lateral

## Abstract

**Background/Objectives:** Lateral spine surgery offers effective minimally invasive deformity correction, but traditional approaches often involve separate anterior, lateral, and posterior procedures. The prone lateral technique streamlines this process by allowing single-position access for lateral and posterior surgery, potentially benefiting from the lordosing effect of prone positioning. While previous studies have compared prone lateral to direct lateral for adult degenerative diseases, this retrospective review focuses on the outcomes of adult deformity patients undergoing prone lateral interbody fusion. **Methods:** Ten adult patients underwent single-position prone lateral surgery for spine deformity correction, with a mean follow-up of 18 months. **Results:** Results showed significant improvements: sagittal vertical axis decreased by 2.4 cm, lumbar lordosis increased by 9.1°, pelvic tilt improved by 3.3°, segmental lordosis across the fusion construct increased by 12.2°, and coronal Cobb angle improved by 6.3°. These benefits remained consistent over the follow-up period. Correlational analysis showed a positive association between improvements in PROs and SVA and SL. When compared to hybrid approaches, prone lateral yielded greater improvements in SVA. **Conclusions:** Prone lateral surgery demonstrated favorable outcomes with reasonable perioperative risks. However, further research comparing this technique with standard minimally invasive lateral approaches, hybrid, and open approaches is warranted for a comprehensive evaluation.

## 1. Introduction

Adult spinal deformity (ASD) is a debilitating condition with a significant effect on quality of life. However, surgery can improve radiographic parameters and patient-reported outcomes (PROs) in select patients with ASD [1]. Traditional open techniques are effective but are associated with significant blood loss, infection risk, prolonged hospital stays, lengthy recovery periods, and high rates of proximal junctional kyphosis/failure (PJK/PJF) or adjacent segment disease necessitating further surgery [2,3]. Minimally invasive surgery (MIS) techniques such as lateral interbody fusion are associated with less patient morbidity compared to open techniques and are now well established for treating ASD, but achieving significant sagittal radiographic correction, as in cases of iatrogenic “flatback” or severe sagittal imbalance, remains challenging [4]. The reasons for this continue to be investigated, and among them are the inability to carry out powerful closing osteotomies and the fact that the segmental correction achieved in the lateral position does not appear to translate significantly to overall lumbar lordosis (LL) [5]. This has led to “hybrid” approaches with staged MIS anterolateral and open posterior work, including osteotomies.

Recently, there has been an increased interest in the prone lateral interbody technique, which may represent a more efficient method for carrying out anterolateral surgery and prone surgery without needing to ‘flip’ the patient. Prone lateral interbody fusion has been shown to provide greater segmental lordosis (SL) than the lateral decubitus position, likely because of gravity assistance from prone positioning [6]. It has also been suggested to give greater gains in LL for degenerative patients [6,7]. Another cited strength of prone lateral is that two surgeons can work simultaneously [6,8,9]. Furthermore, recent work has supported the safety of prone lateral surgery [10].

From a radiographic perspective, a multi-institutional retrospective study by Theologis et al. found that prone lateral could offer better correction of major Cobb angles, lumbar pelvic parameters, and sagittal vertical axis (SVA) than posterior-only MIS operations [11]. While these reports mainly describe adult degenerative conditions with a limited number of levels addressed, the literature investigating the use of prone lateral methods for the treatment of ASD is limited [12,13]. The objective of this study, therefore, was to evaluate the utility of the prone lateral approach in the treatment of adult deformity. We aim to understand if the benefits of prone lateral interbody fusion surgery described in degenerative conditions can be applied to spine deformity.

## 2. Materials and Methods

### 2.1. Patient Selection

This is a single-surgeon retrospective review of ASD patients at a large tertiary care center who underwent a prone lateral approach between 2020 and 2022. Patient inclusion criteria included age ≥ 18 and characteristics of spinal deformity as defined by spinopelvic parameters. We defined this as preoperative SVA ≥ 6 cm, pelvic incidence to lumbar lordosis (PI-LL) mismatch ≥ 10, and/or coronal Cobb angle ≥ 20°.

### 2.2. Technique

**Figure 1:**
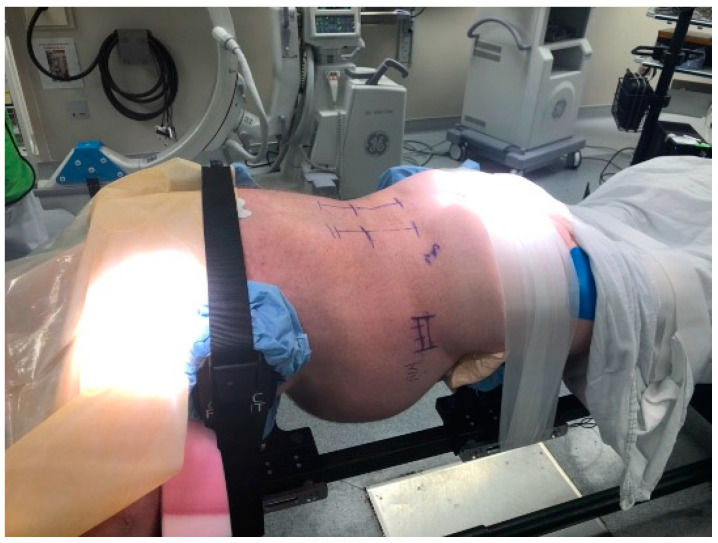
Patient positioning for the prone lateral approach. The patient is positioned prone with arms in a “Superman” position. The hip pads are positioned slightly lower to accentuate lumbar lordosis. It is important to have the patient securely taped into position to provide adequate oppositional forces while placing trials/cages. Others have described the use of special coronal benders to aid in access to L4-L5, especially when there is a high iliac crest.

The surgical technique has been previously described by Pimenta et al., and our modified technique has been described by Wang et al. [9,13]. Patients undergoing prone lateral interbody fusion for deformity are positioned prone in an open Jackson frame with the arms in the “Superman” position, the lower extremities extended, and the abdomen hanging freely. Under fluoroscopy, the length of the disk space is marked, and the posterior incision is planned as per surgeon’s preference. We use real-time virtual live fluoroscopy (TrackX Technology, Hillsborough, NC, USA) for instrument guidance and confirm implant and instrumentation placement with intraoperative CT. The detailed patient positioning is shown in Figure 1.

For the lateral incision, the retroperitoneal space is accessed via dissection down to the muscle fascia. No additional retroperitoneal incision is necessary. Once the psoas muscle is exposed, an initial dilator with an integrated wire is inserted into the lateral opening and docked in the middle of the disk space. Once the positioning is confirmed with fluoroscopy, the wire is introduced into the disk space, and serial dilation with circumferential electromyographic (EMG) stimulation is performed, using 8 mA as the cutoff for retractor placement. The retractor is then fixed over the final dilator and expanded anteriorly and posteriorly along the disk space. The wire is then removed and the surgeons proceed with discectomy assisted by fluoroscopy, straight and angled Cobb, and ring curettes. After performing trials to find the appropriate implant, the final implant is put in place. Posterior instrumentation can then be exchanged, and new instrumentation put in place for posterior fixation or decompression.

### 2.3. Outcomes

Demographic information collected included age, sex, body mass index (BMI), smoking status, and whether the patient had diabetes, osteoporosis, or had undergone previous spine surgery. Perioperative measures included number of surgical levels, laterality of approach, operative time, estimated blood loss, intraoperative complications, and dose of fluoroscopy, while postoperative variables included length of hospital stay, 30-day readmission, and complications. Radiographic outcomes included changes in global alignment, as measured by changes in SVA, PI-LL mismatch, and coronal Cobb angle, as well as segmental changes in lordosis. We also measured PROs including the Oswestry Disability Index (ODI) and Visual Analogue Scale (VAS). Both PROs and radiographic outcomes were collected preoperatively and six weeks postoperatively for all patients. Additional follow-up PROs and radiographic outcomes were recorded for later postoperative visits, as available.

### 2.4. Statistical Analysis

Univariate analyses were performed using one-sample *t*-tests. Pre- and postoperative radiographic measurements and patient-reported outcome measures were analyzed using paired *t*-tests. To assess long-term clinical outcomes, Spearman rank coefficients were utilized to assess possible relationships between changes in radiographic measurements and PROs. We utilized ANOVA to quantify the effects on radiographic measurements and clinical outcomes of the prone lateral approach compared to hybrid and circumferential minimally invasive surgery (MIS) approaches previously found in the literature [14]. For all analyses, a *p*-value of less than 0.05 was considered statistically significant.

## 3. Results

### 3.1. Demographic Information

Ten patients underwent deformity correction, with a mean follow-up of 16 months. The cohort was 70% male, with a mean age of 67.5 (5.8) and mean BMI of 30.4 (4.6) kg/m^2^ (Table 1). Two (20%) patients had co-morbidities of osteoporosis and one (10%) of diabetes. All were optimized, with two receiving a course of anabolic bone agents and the diabetic patient had hemoglobin A1C under 6.5. Two (20%) were smokers, who, despite counseling and briefly quitting, resumed smoking after surgery. Four (40%) had a previous instrumented spine fusion. Average skin-to-skin time was 569 (158) minutes with 316 (97) mGy radiation dose (Table 2). Eight out of ten patients (80%) had a left-sided approach, with the others having a right-sided one, due to patient anatomy. Eight patients (80%) had static cages, with two using expandable cages. Seven out of ten patients (80%) also underwent an ALIF at L5-S1 and one underwent L4-S1 ALIF. The mean estimated blood loss and length of hospital stay were 1010 (723) mL and 7.3 (5.5) days, respectively. Each case averaged 4.9 levels (range: 2–9).

Three (30%) patients had a perioperative complication and none had readmissions within 90 days. Of these complications, one patient required revision surgery at 17 months post-op for PJF, one had ileus with prolonged hospital stay, and one patient had sacroiliac joint pain requiring an injection in the perioperative period. Two (20%) of the patients experienced short-term hip flexor weakness.

### 3.2. Radiographic Outcomes

**Figure 2:**
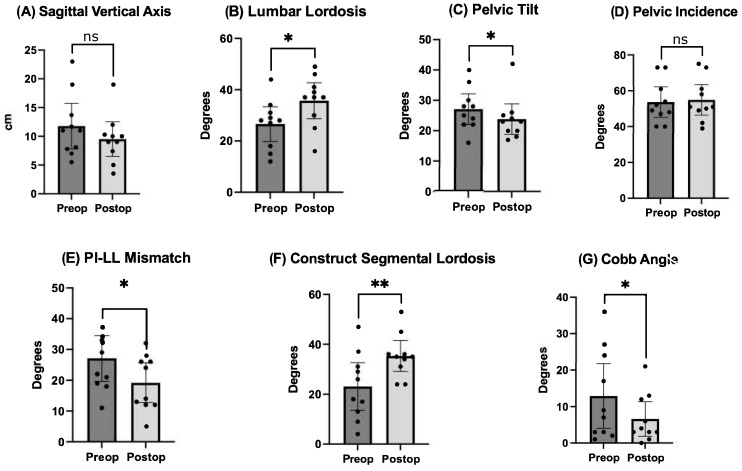
Comparison of pre- and postoperative radiographic outcomes of spinopelvic parameters. Undergoing prone lateral deformity surgery was associated with significant improvements in lumbar lordosis, pelvic tilt, PI-LL mismatch, SL across the construct, and coronal Cobb angle. * = *p* < 0.5, ** = *p* < 0.01, ns = nonsignificant.

**Figure 3:**
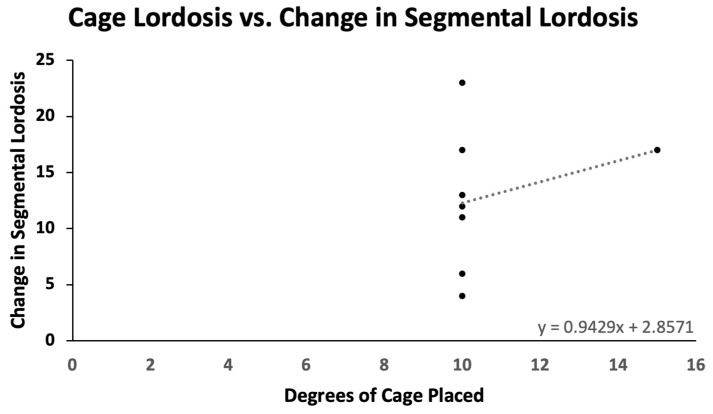
Cage lordosis versus change in segmental lordosis. For every additional degree of cage placed, there is an average gain of 0.943° in segmental lordosis.

**Figure 4:**
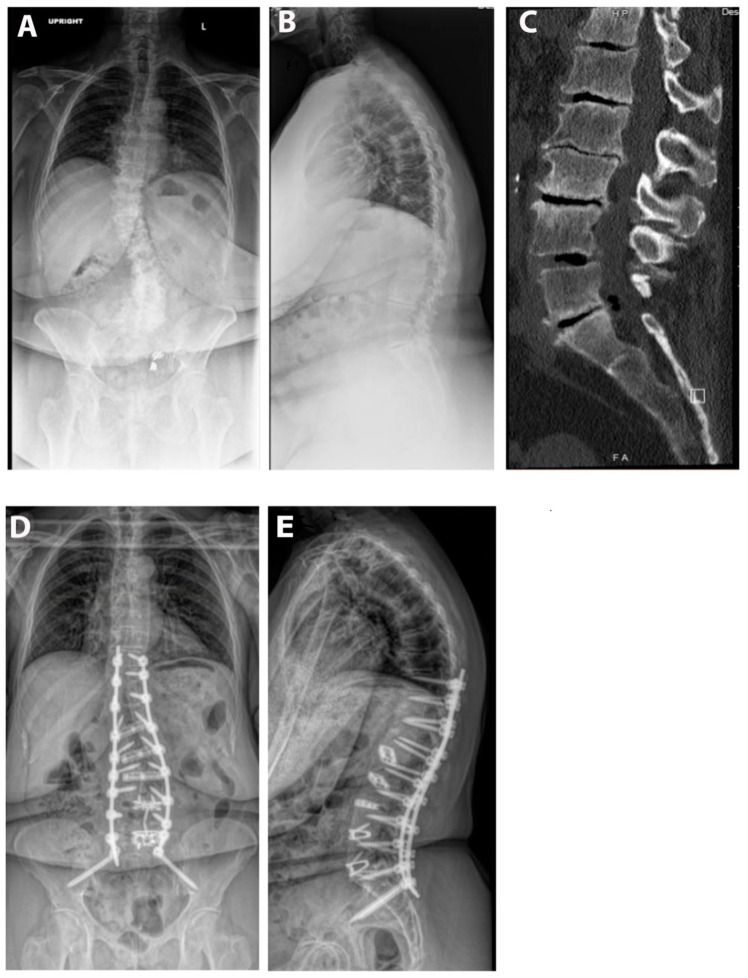
Example case. The patient is a 67-year-old male with a BMI of 36. He was having severe back and leg pain and had attempted extensive conservative measures. His pre-op SVA was 11 and PI-LL mismatch was 35°. He underwent an L4-S1 ALIF and then a L2-5 prone lateral with T10-pelvis posterior fusion. (**A**) Pre-op upright AP X-ray. (**B**) Pre-operative upright lateral X-ray. (**C**) Pre-operative CT scan. (**D**) AP postoperative upright AP X-ray. (**E**) Postoperative upright lateral X-ray.

Pre- and postoperative measurements were made of spinopelvic parameters from full-length standing X-rays (Figure 2 and Table 3). Postoperative measurements were taken at the last follow-up appointment. These patients had a mean SVA pre-operatively of 11.8 (range: 5.5 to 23), and on average improved by 2.4 cm (range: −0.7 to −4.7, *p* = 0.15). Mean pre-op PI-LL mismatch was 27.1 (range: 11 to 42) and this decreased significantly by 7.9° (range: 5 to 32, *p* = 0.01). Lumbar lordosis (LL) increased significantly, on average by 9.1° (range: −2 to 31, *p* = 0.01) after surgery, and the mean change in pelvic tilt (PT) was 3.3° (range: −13 to 4, *p* = 0.04). The mean coronal Cobb angle was 12.9° preoperatively (range: 1 to 36), with a significant mean postoperative decrease of 6.3° (*p* = 0.03). The average change in segmental SL was +12.2° (range: +4 to +23, *p* = 0.004). On average we placed 10.63° (range: 10–15) of cages in these patients (Figure 3). Of note, only one patient had a posterior column osteotomy. An example case of a patient who underwent an L5-S1 ALIF with L2-5 prone lateral interbody fusion is shown in Figure 4.

On follow-up, no additional patients underwent additional corrective surgery or exhibited evidence of PJK/PJF. Additional full-length standing X-rays were available for seven patients, with a mean follow-up time of 23.6 months. In these patients there were no significant differences between the spinopelvic parameters in their most recent imaging compared to those in their initial postoperative imaging.

### 3.3. Clinical Outcomes

The mean preoperative VAS score was 6.9 (range: 5 to 9) and decreased by three points postoperatively (*p* < 0.001). Pre- and postoperative ODI scores were available for six out of ten patients. The average change in ODI scores was 11.57 (range: 0 to 18) from the preoperative mean of 29.1 (*p* = 0.007). Analysis of Spearman rank coefficients revealed a strong positive association between reductions in ODI scores and improvement in SVA (Table 4). Moderate negative and positive associations were observed between both VAS and ODI scores, respectively, and improvements in SL.

### 3.4. Comparative Analysis

Comparing the prone lateral approach to the circumferential MIS and hybrid approaches, we found a statistically significant difference in the mean change of radiographic outcomes in both SVA (*F*(2) = 3.7926, *p* = 0.024) and coronal Cobb angle (*F*(2) = 18.1661, *p* < 0.001), by type of surgery. Tukey post hoc testing revealed that the prone lateral approach yielded greater improvements on average in SVA compared to the hybrid approach (2.67 cm, *p* = 0.04). There was also evidence that the hybrid approach resulted in greater changes in coronal Cobb angle on average than the prone lateral approach (14.6°, *p* < 0.001). For clinical outcomes, there were statistically significant changes in the mean VAS (*F*(2) = 25.658, *p* < 0.0001) by surgery type when comparing follow-up data. Post hoc testing showed that the prone lateral approach resulted in greater improvement compared to the MIS approach for ASD (5.8, *p* < 0.0001) (Table 5).

## 4. Discussion

This study suggests that the prone lateral approach is safe and effective in the treatment of ASD. To the authors’ knowledge, this is the largest reported case series describing outcomes for the prone lateral approach on the subject. Our findings support the fact that the prone lateral approach can achieve a statistically significant increase in LL and reductions in PI-LL mismatch and coronal Cobb angle, as well as significant improvement in SVA and PT. These results are consistent with both the prone lateral degenerative spine literature and with few reports of deformity [15]. We also show significant reductions in both VAS and ODI scores, which were correlated with improvement in SVA and SL. In comparison to the existing literature on surgical approaches to spine deformity, we found that the prone lateral approach yields greater improvements in SVA compared to hybrid approaches.

From a perioperative standpoint, we had a relatively low complication rate, with no “major” complications as defined by Fritzell et al. and only one re-operation at 17 months post-op [16]. This compares to rates of 20–30% in open deformity. The recent literature on ACR shows a similar total complication rate if transient psoas or quadriceps weakness is included [17,18]. EBL and length of stay in this series were also comparable to lateral decubitus ACR and favorable to open deformity techniques.

It is also of note that nine of our ten cases were fusions to S1 rather than the pelvis. It has been suggested that “long construct” posterior fusions should be anchored by pelvic instrumentation, but whether pelvic fixation is required with the upper instrumented vertebrae of L2, which was the case for most of our patients, is unclear [19]. There is known morbidity related to pelvic fixation, including screw loosening and prominence requiring revision [20]. Future work should measure long-term outcomes for these fusions stopping at the sacrum.

We achieved an SL increase of 12.2° while placing 10.63° (10,15) of cages in patients with an average pre-op SL of 23.1°. The amount of long-term SL gained from static and expandable interbody cages has been debated. Bakare et al. showed that for 10 and 12° lateral cages, the SL at the last follow-up was 13.9° and 18.7°, respectively [5]. Lovecchio et al. reported a gain of 4.5° per lateral level with a 15° cage [21]. Though these series were not seen in deformity patients, they are consistent with our findings [22].

In terms of clinical outcomes, we showed significant decreases in both VAS and ODI postoperatively, along with moderate-to-strong associations between PROs and SVA and SL. In addition, when compared to MIS and hybrid techniques for ASD correction, prone lateral was superior to the MIS approach in decreasing VAS. As prone lateral is a relatively novel approach, there is a paucity of literature on long-term clinical outcomes of the technique, especially for ASD. However, previous studies of patients undergoing fusion for ASD in general have shown strong correlations between improvement in radiographic measurements and clinical outcomes such as ODI and VAS consistent with our study [23,24]. In contrast, a 2017 study on ASD and patient satisfaction showed that although VAS and ODI were associated with improved patient quality of life, this was not associated with improvement in radiographic measures, citing the fact that the patient–surgeon relationship, surgery goals, and setting appropriate preoperative expectations may be more important [25]. Although our study lays the groundwork for the improvement in clinical outcomes after prone lateral interbody fusion, further work should focus on the degree of patient satisfaction in addition to PROs following prone lateral for ASD correction.

While prone lateral can achieve statistically significant SL correction in deformity patients, we are not achieving a correction similar to pedicle subtraction osteotomy (PSO) or ACR, which can yield 25–35°, along with greater SVA correction [26,27]. However, performing a three-column osteotomy or large ACR is associated with significant complications, including neurological injury, vascular injury, and durotomy [20,28,29]. Further correction can be gained by performing posterior column osteotomies in a “hybrid” MIS approach, as shown in Chan et al. [14]. However, the patients in this series were “true” MIS cases, with 100% percutaneous screws. Although we found some evidence that the prone lateral approach may improve radiographic measurements compared to hybrid MIS, future work should aim to further characterize these differences to optimize deformity correction while mitigating postoperative complications.

In 2019, Mummaneni et al. described MISDEF2, an updated algorithm for patient selection in minimally invasive deformity, which characterizes four classes of patients with increasing complexity [30]. Class I patients do not have significant radiographic deformity and can be treated with MIS surgery. Class II patients have moderate radiographic deformity and can be treated with multi-level MIS surgery. Class III patients have severe radiographic deformity and are best treated with circumferential MIS surgery, sometimes with anterior column reconstruction or hybrid posterior open approaches with osteotomies. Finally, class IV patients have rigid spines and over 5 levels, requiring open fusion surgery to address severe deformity. In our series, we had one class II and nine (90%) class III patients. We propose that prone MIS approaches should be considered to efficiently deal with class III patients or be incorporated in class IV patients who would benefit from anterior column support. For deformity patients who strongly prefer, or are more medically suited to, MIS surgery, prone lateral may be an advantageous option. With the average patient age increasing, less-aggressive deformity correction may be needed, based on the concept of an age-adjusted alignment target [31]. Finally, from an efficiency standpoint, prone lateral has the potential to decrease operative time when two operators are available or when there are institutional issues with patient-positioning time. Increased OR time has been shown to correlate with complications and patient outcomes, and deformity corrections are often lengthy surgeries, whether open or MIS [32]. Supported by our series as well as several other reports, the prone lateral approach is associated with improved patient VAS and ODI and a reduced complication profile compared to other corrective surgeries [7,10]. Being able to efficiently place anterolateral interbody support while placing percutaneous screws or performing “mini open” or open osteotomies in complex-deformity patients with stiff spines (e.g., MISDEF2 class IV patients) would be a technical advancement in spine surgery, with greater benefit to patients.

Our study has several important limitations. Most importantly, it is a retrospective review from a single institution with a limited sample size. Furthermore, while our average follow-up was over one year, for deformity patients it is likely that longer-term follow-up is needed to truly appreciate complications and long-term radiographic outcomes [33,34]. There are several caveats from a technical perspective as well. This study does not address ACR, a potentially powerful MIS deformity-correction technique shown to significantly improve SVA and LL [4,35]. Current prone MIS techniques also have the issue of not being able to access L5-S1 without repositioning, whereas direct lateral or anterior to psoas (ATP) techniques can provide single-position access. L5-S1 is perhaps the most important single level for restoring lordosis in deformity correction, as evidenced by our study where 80% of patients also underwent supine ALIF [36,37]. Finally, prone lateral is a newer and less familiar technique for many spine surgeons. While lateral surgery is growing in popularity, only a subset of lateral surgeons thus far have adopted prone lateral surgery.

## 5. Conclusions

In our series, prone lateral provides improved SL, LL, and improved SVA and PT for deformity patients, along with improved PROs. We also achieved SL gains greater than the degrees of cage placed, which may be due to the assistance of gravity in the prone position. The correction achieved is less powerful than techniques such as PSO and ACR, but is a safe and reproducible technique. It is challenging for MIS techniques to achieve sagittal plane correction on par with open deformity surgery, and we do not yet know the long-term outcomes of patients who are “under-corrected” by traditional deformity parameters but who are doing well clinically. Increasingly, patients are older and with more comorbidities, and with the known benefits of MIS surgery it is important to continue innovating techniques to achieve similar or better functional and radiographic outcomes than with open deformity surgery. Prone lateral attempts to mitigate the invasiveness of traditional approaches while achieving similar outcomes. The growing literature suggesting prone lateral techniques lead to improved lordosis along with other benefits such as surgeon ergonomics and decreased operative time deserves further investigation for an obvious application—deformity surgery. Prone lateral approaches can decrease OR time or improve lordosis in adult degenerative cases, especially after surgeons progress along a learning curve, but the benefits for a short-segment fusion case would be less than for a complex deformity. We look forward to larger prospective studies with long-term outcomes and a better understanding of prone MIS techniques for spine deformity.

## Figures and Tables

**Table 1:** Demographic Information.

Demographic	Mean (SD)
Sex	70% Male, 30% Female
Age (Years)	67.5 (5.8)
BMI (kg/m^2^)	30.3 (4.6)
Active Smoking Status	20%
Diabetes	0%
Osteoporosis	0%
Previous Surgeries	20% Lumbar Decompression 20% Posterior Spinal Fusion

**Table 2:** Perioperative Metrics.

Metrics	Mean (SD)
Number of Operative Levels	4.0 (1.5)
Number of Lateral Levels	2.2 (0.6)
Direction of Approach	80% Left, 20% Right
Skin-to-Skin (min)	569 (158)
Fluoroscopy Dose (mGy)	316 (97)
Estimated Blood Loss (mL)	1010 (723)
Length of Hospital Stay (days)	5.7 (2.2)
Complications (n patients)	Constipation (1) SI Joint Pain (1) Transient Weakness (1)

**Table 3:** Pre- and Postoperative Outcomes of Radiographic Spinopelvic Parameters.

Radiographic Parameter	Baseline Mean (SD)	Postop Mean (SD)	Change Mean (SD)	*p*
Sagittal Vertical Axis (cm)	11.8 (5.5)	9.5 (4.2)	−2.4 (2.5)	0.15
Lumbar Lordosis (°)	26.6 (9.5)	35.7 (9.8)	+9.1 (9.6)	0.01
Pelvic Tilt (°)	27.1 (7.0)	23.8 (7.3)	−3.3 (4.5)	0.04
Pelvic Incidence (°)	53.7 (11.9)	54.9 (12.0)	1.2 (5.0)	0.27
PI-LL Mismatch (°)	27.1 (10.4)	19.2 (9.0)	−7.9 (9.8)	0.01
Construct Segmental Lordosis (°)	23.1 (12.6)	35.3 (8.3)	+12.2 (8.8)	0.004
Cobb Angle (°)	12.9 (12.4)	6.6 (6.7)	−6.3 (9.4)	0.03

**Table 4:** Spearman Rank Correlations between Patient-Reported Outcomes and Radiographic Measurements.

Change in Radiographic Parameter	Change in PROs	
	ODI	VAS
Sagittal Vertical Axis (cm)	−0.5778	0.1274
Lumbar Lordosis (°)	−0.4772	0.3039
Pelvic Tilt (°)	−0.3069	−0.3377
PI-LL Mismatch (°)	−0.4772	0.3039
Segmental Lordosis (°)	−0.4421	0.4469
Cobb Angle (°)	−0.1383	−0.0765

**Table 5:** Comparative Analysis of Prone Lateral, MIS, and Hybrid Approaches to ASD.

Radiographic Parameter	Prone Lateral	MIS	Hybrid	F	*p*
Sagittal Vertical Axis (cm)	−2.4 (2.5)	−0.59 (0.6)	0.27 (4.9)	3.7926	0.02
Pelvic Tilt (°)	−3.3 (4.5)	0.6 (7.1)	−0.1 (7.9)	1.3467	0.26
PI-LL Mismatch (°)	−7.9 (9.8)	−3.8 (14.7)	−6.8 (16.8)	1.0672	0.35
Cobb Angle (°)	−6.3 (9.4)	−14.5 (2.6)	−20.9 (13.6)	18.1661	<0.0001
ODI	−11.6 (6.3)	−21.1 (21.1)	−19.3 (18)	0.7865	0.4568
VAS	−3.0 (1.4)	−2.8 (2.9)	−3.6 (2.8)	25.6580	<0.0001

## Data Availability

The data presented in this study are available on request from the corresponding author.
